# Exploring mediators of the recovery process over time among mental health service users, using a mixed model regression analysis based on cluster RCT data

**DOI:** 10.1186/s12888-020-02924-2

**Published:** 2020-10-30

**Authors:** Elisabeth Argentzell, Martin Bäckström, Kristine Lund, Mona Eklund

**Affiliations:** 1 Lund University, Department of Health Sciences, the Mental Health, Activity and Participation (MAP) group, Box 157, 221 00 Lund, Sweden; 2 Lund University, Department of Psychology, Box 213, 221 00 Lund, Sweden

**Keywords:** Recovery, Mental illness, Activities of daily living

## Abstract

**Background:**

Personal recovery is associated with many significant health-related factors, but studies exploring associations between activity factors and personal recovery among service users are scarce. The aims of this study were hence to; 1) investigate if various aspects of activity may mediate change in recovery while also acknowledging clinical, sociodemographic and well-being factors; 2) explore the effects of two activity-based interventions, Balancing Everyday Life (BEL) or standard occupational therapy (SOT), on personal recovery among service users.

**Methods:**

Two-hundred-and-twenty-six service users were included in a cluster RCT, 133 from BEL units and 93 from SOT units. Participants commonly had a diagnosis of mood disorder and the mean age was 40. Instruments used targeted activity, mastery and functioning. A mixed-model regression analysis was employed.

**Results:**

The model tested was whether selected variables could be used to mediate the change in recovery from the start to a six-month follow-up after intervention. Participants’ personal recovery increased after treatment and increased further at the follow-up. The general level of recovery was negatively related to a diagnosis of depression/anxiety, both before and after treatment, but depressed/anxious service users still increased their recovery. There were no significant relations between recovery and sex or age. The interactions between change in recovery and changes in depression/anxiety, satisfaction with activities, sex, and age were all non-significant. All possible treatment mediators included were related to change in recovery, the strongest being occupational engagement and mastery, followed by activity satisfaction and symptoms. Mediation was shown by the decrease in the effect of the time factor (from intervention start to completion) when the covariates were introduced. In all cases the time variable was still significant. When testing a model with all variables simultaneously as covariates, occupational engagement and mastery were strongly significant. There was no difference between interventions regarding recovery improvement.

**Conclusion:**

The treatments were equally beneficial and were effective regardless of gender, age and diagnosis. Those who gained most from the treatment also gained in feelings of mastery and activity engagement. Activity engagement also moderated the level of recovery. To enhance recovery, interventions should facilitate meaningful activities and gaining control in life.

**Trial registration:**

The study was registered with ClinicalTrials.gov. Reg. No. NCT02619318. Retrospectively registered: December 2, 2015.

## Background

The recovery paradigm has increasingly become a central focus within psychiatric care in many western countries, including Sweden [1]. Although the recovery concept is considered important to incorporate within mental health, there is still no consensus regarding factors that may mediate change in recovery scores. Qualitative studies show that various aspects of activity are of importance for the recovery process [2]; however, quantitative studies exploring various aspects of activity that may mediate change in recovery are lacking.

Recovery is a complex and multidimensional concept, which has been defined in various ways [3]. The most prominent two types of definitions that can be differentiated are those of clinical and personal recovery. Clinical recovery adheres to a medical view with a primary focus on objective symptom reduction [4]. Personal recovery, on the other hand, is a subjective ongoing process that involves finding new meaning and hope in life and to be able to engage with others in the community. Personal recovery has been defined as an active and non-linear journey of living a meaningful everyday life with personal growth even with lasting psychiatric symptoms [5]. Personal recovery has been conceptualized as complementary to clinical recovery as it represents a personal process more than a defined outcome. Furthermore, studies have shown that sole reduction of symptoms (i.e. clinical recovery) does not necessarily result in higher personal recovery [6, 7]. Lim et al. [8] suggest that since it is a subjective view from the people themselves, personal recovery offers a more holistic definition of the recovery concept. They contend that personal recovery be the primary focus when evaluating recovery in mental health services, although clinical recovery may also be considered as a supplement [8].

In order to provide a theoretical framework for investigating factors that can promote personal recovery, Leamy and collegues [9] conducted a systematic review and narrative synthesis of recovery research. The review highlighted the concepts of Connectedness, Hope and optimism, Identity, Meaning and Empowerment, forming the CHIME framework, as paths leading to personal recovery. CHIME currently is one of the most prominent and well-used frameworks for personal recovery in mental health services and research [10], including the current study. The choice of this specific framework was also based on the fact that the different factors in CHIME possibly interact with activity engagement [2], although this needs to be further investigated.

Personal recovery has been viewed as an important outcome measure in mental health care, and in order to identify important targets in rehabilitation work, a number of quantitative studies have investigated whether personal recovery is associated with other health factors. For example, it has been indicated that a higher level of quality of life is strongly associated with better recovery [3, 11, 12] and that the strongest longitudinal factors in explaining variation in recovery were psychosocial factors and negative emotion [6]. A further study [13], showed that increased recovery was correlated with certain sociodemographic factors such as being female and having a higher income. Additionally, higher levels of internal locus of control have been shown to be associated with higher levels of recovery [14] as well as social support [15]. A recent study by Treichler and colleagues [16] suggested empowerment and consumer involvement to be key components of recovery**.**

Activity engagement cultivates meaning in life for people with psychiatric disabilities [17], which has generated an interest in activity-based approaches within mental health care and research, including investigation of recovery from an activity perspective. The term ‘activity’, as used in this study, is seen as an umbrella concept that covers many aspects of activity. The personal experience of participating in an activity forms a subset of activity aspects, and it is mainly personal experiences such as meaning, satisfaction and level of engagement that are at target in the present study. So far, a number of qualitative studies have studied the role of activity in recovery. A scoping review by Doroud and colleagues [2], based on mostly qualitative studies, explored how activity engagement and recovery were interrelated. The authors recognized personal recovery as a form of ongoing activity process that seemed to include a gradual re-engagement in activity towards more full community participation. The study showed that engaging in meaningful and valued occupations seemed to support recovery through fostering the CHIME factors of connectedness, hope, identity, meaning, and empowerment. This was accomplished by establishing structured routines and assisting people in managing their illness. The authors concluded that activity engagement is an important dimension of the recovery. One of the most prominent articles in the scoping review is an article by Sutton and colleagues [18]. These authors explored the experience and meaning of activity engagement with service users who self-identified as being in recovery from mental illness. The results showed that activity engagement, and also forms of disengagement, played an important role in the recovery process. They also conclude that users’ knowledge of the different modes of engagement and the meaning they could bring were important for clinicians to understand in order to be able to support clients’ recovery journey. A more recent study [19], using the CHIME framework to analyze the daily life of people with severe mental illness, found that everyday activity experiences, within and beyond mental health care, shape personal recovery processes [19]. Few studies have investigated statistical associations between activity factors and recovery, but a quantitative study by Eklund and Tjörnstrand [20] found that personal recovery was associated with satisfaction with daily activities as well as activity level.

Although recovery and activity engagement seem to be related phenomena, interventions targeting activity engagement and recovery are still few within psychiatric care [21]. This focus is however important as many service users have limited activity engagement in their daily lives. One of the existing interventions targeting both activity engagement and recovery for service users is the activity-based occupational therapy program Balancing Everyday Life (BEL). The target group were people with mental illness receiving care in outpatient mental health services: psychosis units, general psychiatric units, and community-based psychiatry. A common selection criterion was that they needed help to counteract imbalance between their activities of everyday life.

The intention with the BEL intervention was that the participants would develop an ability to reflect on their own situation and gain strategies for accommodating their activity engagement, such that they would increase their satisfaction with everyday activities and thereby possibly also their personal recovery. They also got training in understanding the recovery concept. BEL contrasts against interventions that address recovery more specifically since it has a strong focus on activity engagement. The program has been evaluated in an RCT [22] showing positive effects on activity engagement, activity level and activity balance, as well as on symptom severity and level of functioning. The BEL group also improved their quality of life more than the comparison group from baseline to a follow-up. Qualitative studies from that project show that the intervention seemed to enhance activity and key recovery tenets over time [23, 24]. This raises questions about the intervention’s impact on the participants’ personal recovery, as well as the importance of activity-oriented factors for improved personal recovery and how they might potentially mediate recovery over time.

Taken together, personal recovery has been associated with many significant health-related factors and socio demographic factors. Although activity engagement has been shown to be of importance for the target group’s recovery in a number of qualitative studies, few studies have explored associations between activity factors and personal recovery over time. Such knowledge is important to refine interventions for mental health service users. Also, the prominent recovery framework CHIME is lacking a dimension of activity as a part of the recovery journey [9]. The aim of this study was hence to investigate if various aspects of activity, such as activity engagement, activity level and activity balance, may mediate and/or moderate change in recovery among persons with mental illness, while also considering clinical, sociodemographic and health-related factors. A further aim was to explore the possible effect of the BEL intervention, compared to standard occupational therapy (SOT), on personal recovery among users of mental health care services. We hypothesized that the targeted aspects of activity would have a positive effect on recovery and that the BEL group would improve more on recovery than the SOT group.

## Methods

This study included people who had participated in an RCT study and consisted of two subsamples; those who had received the activity-based intervention BEL and those who had received SOT [25]. Both samples received psychiatric care according to best practice in community-based mental health care. One also received the manualized BEL program, whereas the other received SOT, either in the context of outpatient mental health services or community-based mental health centers. The study adheres to CONSORT guidelines.

### Selection of settings and participants

**Fig. 1:**
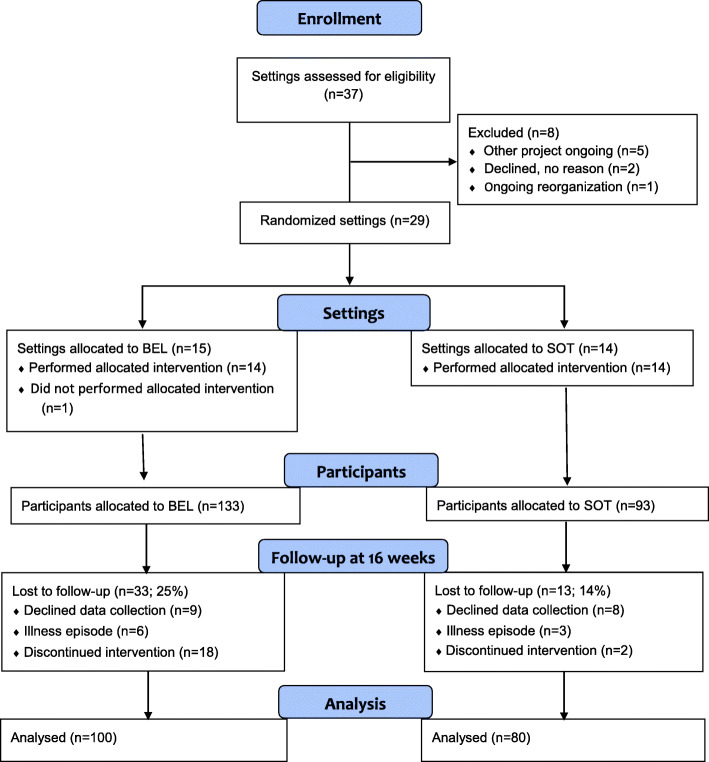
Diagram of inclusion of settings and subjects

The flow of included settings and participants is shown in Fig. 1. All 37 outpatient units within general psychiatry and psychosis care and all community-based mental health centers in three Swedish regions were invited to the project. Eight of these were excluded because of ongoing re-organization or other ongoing projects. Fifteen of the remaining 29 were randomized to the BEL and 14 to the SOT group. One BEL unit then withdrew because the occupational therapist was on sick leave.

An occupational therapist employed at the respective units served as a gatekeeper and identified eligible participants. Inclusion criteria were age 18–65 years, a main diagnosis other than substance use disorder, and no comorbidity of dementia or developmental disorder. This meant that a broad range of psychiatric diagnoses were accepted, including psychoses, mood disorders and neuropsychiatric disorders, but the choice of service context (general psychiatry, psychosis care, community-based mental health centers) entailed that all eligible participants had a major mental health condition. No interpreters were used for the data collection; participants thus also needed to have good command of the Swedish language. All eligible participants were invited, and the gatekeeper provided oral and written information about the project. Those who accepted the invitation were then contacted by a research assistant, and before data collection started, they gave their written informed consent. The research assistant arranged an appointment with each participant, and the data collection then took place in a quiet and private room in the respective settings. Participants received a small reimbursement and had, when relevant, their travel expenses covered.

A total of 226 participants were included; 133 from BEL units and 93 from SOT units. These numbers were somewhat lower than the desired sample sizes based on a power analysis performed on the instrument Satisfaction with Daily Occupations (SDO) assessment [26]. Using the means and standard deviations from a previous study [27] we arrived at 82 participants in each group to detect a difference on the SDO of 0.5 with 80% power at *p* <  0.05. Presuming 25% attrition, we sought to include 120 participants from BEL setting and the same number from SOT settings. Background information regarding the two groups of participants are displayed in Table 1, showing that they were comparable on all known sociodemographic and clinical characteristics.

**Table 1:** Sociodemographic and clinical characteristics of the participants

Characteristics	BEL participants *N* = 133	SOT participants *N* = 93	*P*-value
Gender (% women)	77	67	Ns.
Age (mean, SD)	40 (11)	40 (11)	Ns.
Born in Sweden	93	88	Ns.
Married or cohabiting (%)	30	26	Ns.
Has children living at home (%)	47	47	Ns.
Education (%)			Ns.
Nine-year compulsory school or lower	18	21	
High school	59	60	
College/university education	23	19	
Self-rated diagnosis (%)			Ns.
Psychosis	19	24	
Anxiety/bipolar/depressive disorders	52	50	
ADHD/ADD	23	16	
Other	6	10	
Takes psychotropic medicine (%)	92	93	Ns.

From baseline to 16 weeks there were 33 dropouts (25%) the BEL group and 13 (14%) in the SOT group, which was a statistically significant difference (*p* = 0.047). Reasons for dropping out in the BEL group mostly concerned not completing the intervention, such as attending only the first group session. Dropping out of the study due to not wanting to complete the data collection or having an illness episode affected both groups similarly. Between the 16-week measurement and the six-month follow-up another 11 participants (8%) in the BEL group and 10 (11%) in the SOT group dropped out, which was not a statistically significant difference (*p* = 0.527).

### The interventions

#### Balancing everyday life

The BEL intervention was a group- and activity-based program with 14 sessions, spread over 16 weeks and led by an occupational therapist who had taken a two-day education based on the BEL manual [28]. The first 12 sessions had specific themes relating to everyday life, such as meaningful activity, motivators in life, healthy living, work-related activities, and social activities. Each session contained a brief educational section, discussion of the session theme and preparing for a home assignment after the session. Each session meant analyzing the past and (foremost) the present situation, then setting desired activity goals, and finally finding strategies for how to reach the goals. These strategies were then tested in the home assignment, where a desired activity was performed in a real-life context. Goals and strategies were re-negotiated in the next session, if needed, and then a new home assignment followed. This process of analyzing one’s everyday activities, setting goals, deciding about strategies, testing them, and evaluating the outcome formed a process that was repeated for each session, and this set of processes formed the BEL intervention as a whole. Peer support was encouraged during the program, and the last two sessions were booster sessions where the previous themes were revisited and plans were made for how to sustain accomplished progress. The intention with the BEL program was that the participants would develop an ability to reflect on their own situation and gain strategies for accommodating their everyday life, such that they would increase their satisfaction with everyday activities and thereby their personal recovery.

#### Standard occupational therapy

SOT often included some form of group intervention based on, for example, creative or artistic activities, daily living skills, social skills, or handicrafts. Some occupational therapists offered individual therapy only, but typically based on similar activities. SOT was not limited in time, but for the present study, and to align with the BEL program, 16 weeks were decided as the intervention period followed.

#### Similarity between the interventions

Both BEL and SOT was provided by occupational therapists and were activity-based. Moreover, both BEL and SOT occupational therapists were part of a team that could provide a range of mainstream mental health interventions, such as psychotropic medication and some form of supportive therapy and/or follow-up while following principles for “best practice”.

### Data collection

Data was collected at three measurement points: before the interventions began, after the 16-week intervention period, and 6 months following. Research participants met with data collectors at the location of their received intervention. Arrangements were made for a private room and participants received verbal and written information about the study, including the right to terminate their study participation at any time without giving the reason. Self-report and interview-based instruments were administered, as specified below.

#### Socio-demographic and clinical factors

In order to obtain socio-demographic and clinical information, a self-report questionnaire was created for this project. Questions included gender, age, relationship, educational level, mental health diagnosis and experienced mental health problems. A specialized psychiatrist classified the self-reported diagnosis according to the ICD-10 system, according to a procedure described in Eklund and Sandlund (2012). Research indicates that a diagnosis of depression and/or anxiety is related to negative ratings of subjective well-being factors such as quality of life [29, 30]. In order to see if the same pattern could be found for recovery, the diagnoses were grouped into depression/anxiety versus other diagnoses for the analyses of this study.

#### Activity engagement

The self-report version of Profiles of Occupational Engagement among people with Severe mental illness (POES) is a two-part instrument used to measure activity engagement [31, 32]. In the first part, participants fill out the previous day’s events on a 24-h diary sheet. This includes what occupations they engaged in during the day, if they were alone or with someone else, and how they felt when performing the occupation. In the second part, participants reflect on their report of the previous day and answer eight questions. These include questions such as, “I feel that I do different types of activities every day” and “I feel that I have good routines” as well as “I feel that I have mutual relationships and do things with others.” Each of the eight questions is rated on a five-point scale ranging from does not apply at all [1] and totally applies [5]. The POES instrument has been found to have good internal construct validity and inter-rater agreement engagement [31, 32].

#### Activity level and activity balance

The Satisfaction with Daily Occupations and Occupational Balance (SDO-OB) instrument measures activity level, activity satisfaction, and activity balance [33]. The SDO-OB instrument encompasses four occupational segments: work and study, leisure, home tasks, and care of self. Examples of questions include “I currently have a job” (work segment) and “I engage in leisure activities” (leisure segment). Each question in the different areas have a yes/no option, and the yeses are tallied to create the activity level score (the more yeses, the higher the activity score). The satisfaction questions are rated on a Likert-type scale, where scores range from 1 = worst possible to 7 = best possible satisfaction and the ratings are summarized into a satisfaction score. Each of the four segments also concludes with a balance question and the participant rates if they feel their activities in that segment are way too little (− 2), too little (− 1), just enough (0), too much [1] or way too much [2]. In addition, the instrument concludes with a final question about general activity balance with the same scale. The original SDO, without the balance questions, has shown good internal consistency and construct validity [26]. The balance questions, added later, were tested separately and showed satisfactory construct validity [33].

#### Personal recovery

The Questionnaire about the Process of Recovery (QPR) is a self-report questionnaire that addresses different aspects of personal recovery. These aspects include how the person regards their self-worth, their connection with others, and sense of control. Participants rate how much they agree with the different statements by checking one of five boxes that range from “strongly agree” to “strongly disagree.” The original 22-item questionnaire has two subscales and has shown good internal consistency and reliability, and high construct validity [34]. A 15-item one-factor version was found, however, to have better psychometric properties than the original [35]. For this study the QPR-Swe was used, which is a 16-item one-factor version in the Swedish language with good internal consistency and satisfactory construct validity [36].

#### Self-mastery – a health-related factor

The Pearlin Mastery Scale [37] was used to address a health-related aspect. It is a seven-item scale addressing the degree to which people feel they have power over their life situation. Items are worded in terms of “can solve my problems”, “feel I’m being pushed around”, and “control over things that happen to me”. There are four scoring alternatives, from strongly disagree to strongly agree, and some item scorings need to be reversed such that a higher score always indicates more self-mastery. The Swedish version used in the current study has shown good psychometric properties in terms of reliability and validity [38].

#### Level of functioning

The Global Assessment of Functioning (GAF) instrument [39] consists of two ratings made by a research assistant or other professional. One concerns symptom severity and the other psychosocial functioning. Both ratings are made on an interval from 0 to 100, where a higher score indicates better functioning and less severe symptoms, respectively, and a score of 80 or above indicates good mental health. Reliable GAF ratings require training of the raters [40], and after training the research assistants performing ratings in the current project showed high agreement when rating fictional but realistic video cases. GAF has been used extensively and is considered reliable and valid [41].

### Randomization

The settings were assigned to the BEL or SOT intervention by cluster randomization. During 2012–2015, eligible settings were successively grouped in blocks of four units, from which two were randomized to the BEL intervention and two to SOT. In order to ensure allocation concealment and prevent bias, the randomization procedure was handled by an independent research colleague.

### Blinding

Since blinding was not possible in a cluster design, other actions were performed to counteract bias. As stated above, allocation concealment was ensured, and treatment allocation was not specified to the data collector. Also, efforts were made to treat all participants similarly, such as providing an identical information letter, not revealing whether the treatment was BEL or SOT.

### Data analyses

To meet the study aims, we employed mixed model regression analysis using the SPSS [42] package. There were two levels of data, the first being a within-subject factor that included the estimations at the start, the end, and the follow-up of the treatment. This level was called *time*. The other level was a between-subject factor and included the mediators and moderators to the dependent variable, i.e. recovery. All factors were estimated as fixed factors. To model the within-subject variance we used compound symmetry, which estimates the variability in variance and covariance between time points. We first tested the fixed effect of time and thereafter whether the mediators and moderators influenced this effect. A reduction in effect size for the time variable was interpreted as signs of possible mediation. It is important to remember that all the mediators were covariates on the within-subject level; in other words, the possible mediation was related to the treatment effect (the amount of change in recovery between time points). A significant relation between the mediator and the dependent variable was a sign that the change in the mediator, the covariate, was correlated with a change in the dependent variable. There were two kinds of moderator analyses; we investigated (1) whether, sex, age and depression status at baseline interacted with the development on recovery, and (2) whether the mediators also acted as moderators, e.g. that there was a stronger treatment effect for patients who made improvements on the activity variables.

In the models presented below, all participants who had taken part in the data collection on at least one occasion were included, entailing that the baseline estimates were based on more participants, some of whom dropped out at one or two of the following measurements. We also estimated the same models using deletion of persons for whom data was obtained only at baseline. The results were almost identical, even if power was somewhat lower.

## Results

The model tested in this study was whether a number of variables could be used to mediate the change in recovery from the start of the study to the follow up 6 months after the treatment. The mediators included activity engagement, activity level, activity balance, satisfaction with everyday activity, psychosocial functioning, symptom severity, and mastery.

### Treatment effects on recovery

All variables were correlated (see Table 2). The participants were service users from 37 different units. The random effects (i.e. the variability in intercepts and slope of the units) were tested and it was found that a substantial amount of variance could be attributed to the differences in intercepts of the units (the intraclass correlation was = 0.30, marginally significant at *p* = 0.046). However, this random effect did not influence the fixed effects in any substantial way; we therefore report the fixed effects without including random effects in the models.

**Table 2:** Within person covariates to recovery in models including one covariate at a time

Covariates	F	p	Time F	Time p	M1	M2	M3	N
Activity engagement	206.2	< 0.001	4.5	0.012	54.7	56.8^a^	56.4	199
Activity balance	17.7	< 0.001	23.7	< 0.001	52.7	55.8^a^	57.5^b^	218
Activity level	4.7	=0.031	28.0	< 0.001	52.3	56.0 ^a^	57.5^b^	218
Activity satisfaction	79.7	< 0.001	15.5	< 0.001	53.1	55.9^a^	57.0^b^	218
Functioning	33.1	< 0.001	18.7	< 0.001	53.0	56.1^a^	57.2^b^	221
Symptoms	47.6	< 0.001	22.5	< 0.001	52.9	56.1^a^	57.3^b^	221
Mastery	177.2	< 0.001	13.3	< 0.001	53.5	55.6^a^	57.1^b^	222

Note: M = adjusted mean of recovery, M1 = at the start, M2 = at the end, M3 = at follow up; ^a^ = M2 > M1 *p* <  0 .05, ^b^ = M3 > M1 *p* < 0.05

One aim of the study was to compare BEL with SOT. No significant main effect, F (1,214.5) = 0.154, *p* > .05 and no interaction, F (2, 348.6) = 0.151, *p* >  0.05, was found. In other words, there was no difference between the two therapy methods in regard to recovery improvement.

First, the model was estimated without the covariates, and it was found that the change was significant F (2, 351.6) = 30.54, *p* <  0.001; the mean of recovery increased from 52.45, to 56.09, (*p* <  0.001), and then to 57.68 (*p* <  0.001) In addition, the difference between measurement 2 (M2) and measurement 3 (M3) was significant at *p* <  0.05. To summarize, service users rated their level of recovery as better after treatment and as further improved at the follow up.

The general level of recovery was related to a diagnosis of depression/anxiety, F = (1, 213.1) = 14.19, *p* <  0.001. Both before and after treatment, service users with depression/anxiety reported a lower level of recovery compared to those who had other diagnoses. There were no significant relations between recovery and sex or age. The interactions between change in recovery and depression/anxiety, satisfaction with activities, sex, and age were all non-significant as well. This did not support baseline moderators, e.g. service users with different levels of depression/anxiety all gained about the same amount of recovery.

### Possible mediators and moderators to the treatment effect

The main question asked in this study was whether one or more of the variables set as covariates mediated this change in recovery. Table 2 shows the mixed model estimations when the variables were used as covariates to the change in recovery. The treatment effect on recovery was significant for all tested mediators, and the relationships were in all cases positive. The strongest mediators were activity engagement and mastery, followed by activity satisfaction and symptoms. Somewhat weaker effects were found for activity level, activity balance and psychosocial functioning. We also tested all covariates as moderators, and one of them revealed significant moderation; activity engagement moderated the level of recovery, F (2, 318.6) = 4.38, *p* = .013. Service users with an increased activity engagement at the follow up also had a somewhat better recovery at the follow up, B = .42, t (328.8) = 2.92, *p* = 0.004.

Mediation was shown by the decrease in the effect of the time factor when the covariates were introduced one by one. In all cases the time variable was still significant, suggesting only partial mediation. The next step was to test a model with all variables simultaneously as covariates (see Table 3). Four variables were significant in this model, suggesting possible unique mediation for each of these variables. The strongest effects on recovery were from activity engagement and mastery, while activity level and symptoms showed weaker effects. In this model, when all mediators were included, the change in recovery was not significant F (2, 280.1) = 1.62, and the corrected mean values were 55.4, 56.6, and 56.5 for recovery at the three time points. This suggest that the total effect of an activity-based intervention on recovery could be explained by the mediators.

**Table 3:** Within person covariates to recovery in the model including all covariates

Covariate	*F*	*p*
Activity engagement	59.00	< 0.001
Activity balance	0.001	> 0.05
Activity level	2.83	> 0.05
Activity satisfaction	9.72	< 0.001
Functioning	0.499	> 0.05
Symptoms	4.46	0.035
Mastery	57.70	< 0.001

Note: *N* = 196

### Change in standardized values from start of therapy to follow up

**Fig. 2:**
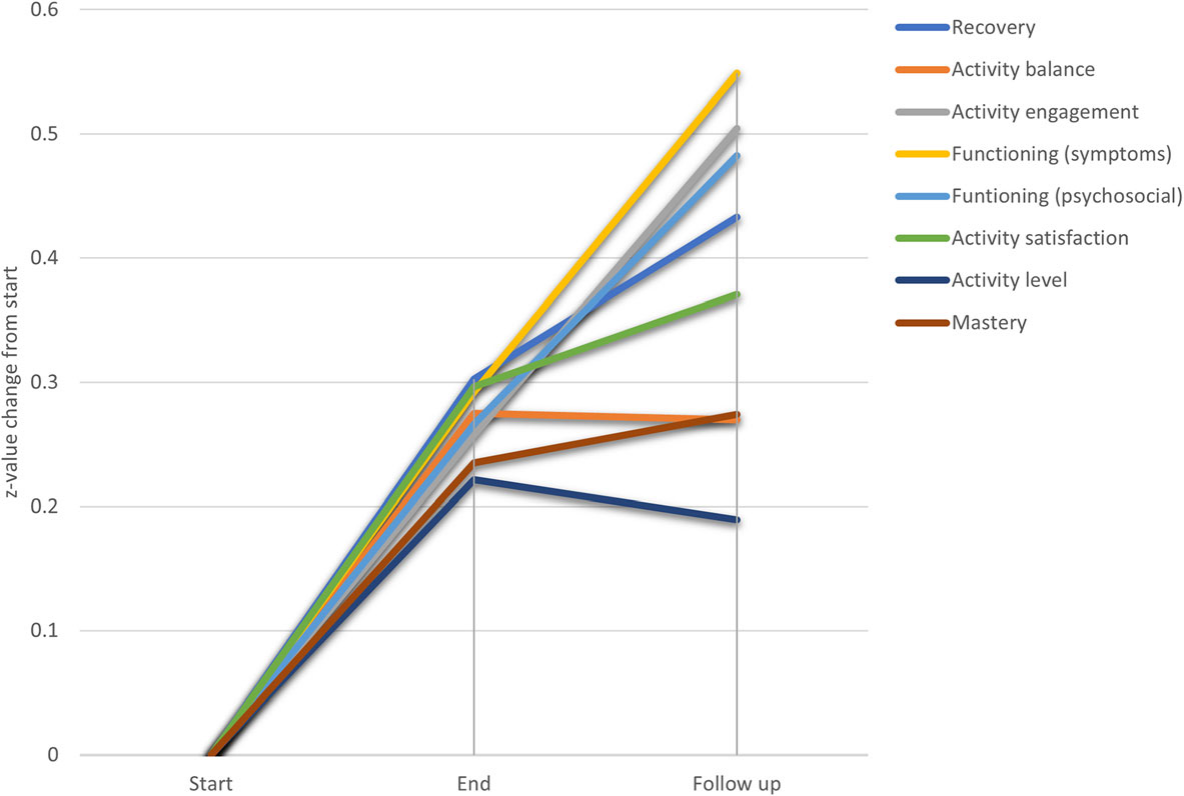
Change in standardized values from start of therapy to follow up

The relative change in standardized units for recovery and all the covariates/mediators are displayed in Fig. 2. As seen there, the recovery variable and all covariates increased steeply between measurements 1 (M1 = before treatment) and 2 (M2 = after treatment). For activity engagement, psychosocial functioning and reduction in symptom severity, there was a linear positive trend over M1, M2 and M3 (follow up). For the other variables, including personal recovery, the increment levelled off to some degree between M2 and M3.

## Discussion

The current study investigated various aspects of activity that could possibly mediate change in recovery, while also acknowledging the influence of clinical and sociodemographic factors and self-mastery, among persons with mental illness receiving activity-based treatments. The results in this study provide novel information about recovery change and mediating factors during treatment for mental health service users. The change per se, from 52.45 at baseline to 57.68 at follow-up on the recovery scale, is an increment of < 10%, suggesting a clinically important change. There was no difference in treatment effect for the two interventions, BEL and SOT. Both types of treatment seemed to have an effect on recovery, but this study also sheds light on possible reasons for this change. With respect to increased recovery, we could not find any support for moderators when addressing gender, age and diagnostic group; the activity-based treatments seem to have been equally beneficial in relation to increased recovery for men and women, and for older and younger clients. These results partly contrast the findings by Tse and colleagues [13], who showed that female service users were more likely than men to have improved recovery scores. However, their recovery instrument differed from the one used in the current study and their study did not look specifically at moderators. The current findings further showed that, although there was a difference in recovery at the start between users diagnosed with depression/anxiety and the other diagnostic groups, and that difference remained over time, the group with a diagnosis of depression/anxiety increased their recovery and benefitted from treatment to the same extent as those without such a diagnosis. That none of these sociodemographic and clinical variables tested as moderators became significant suggests that the activity-based treatments were useful for men and women in various ages and with various diagnoses when aiming at increasing recovery.

The treatment effect on recovery seemed to be followed by effects on the suggested possible mediators. The largest effects were found for mastery and activity engagement, indicating that those who gained most from the treatments in terms of personal recovery also gained in the subjective feelings of mastering one’s life and engaging in different forms of activities in everyday life. That recovery would be affected by mastery is not surprising. Empowerment, a construct with similarities with mastery, is one of the components in the recovery framework CHIME [9]. The finding that increased recovery co-varied with increased engagement in activity is in agreement with both qualitative [2, 19, 43] and quantitative [20] research, suggesting a well-established connection between these phenomena. The relations between recovery and the service users’ reports on activity level and activity balance also align with research showing that creating balance between different everyday activities boosts the recovery process for people with mental illness [17]. Since BEL and SOT are occupational therapy treatments, it is interesting to note that there was a relation between recovery and the extent to which service users were satisfied with their everyday activities. The importance of experiencing satisfaction and finding meaning in activities has been highlighted in recovery literature [9], and research has shown that the recovery process involves moving from disengagement to more engagement in daily life activities [18]. The results from the current study suggest that the CHIME framework would benefit from more clearly highlighting the importance of engaging in satisfying activities in the recovery process.

Moreover, level of symptoms and level of functioning came out as possible mediators in the current study. This is noteworthy, since these variables were not based on the service users’ self-reports but were rated by research assistants. Symptom reduction is generally regarded as clinical recovery, but earlier research has also highlighted that it can be important for personal recovery, though decreased symptoms are not the primary focus for the overall personal recovery journey [8]. As this is in line with our findings, it could be warranted for future research to explore further the relationship between clinical and personal recovery.

The model with all the possible mediators showed that self-mastery and activity engagement contributed uniquely and strongly to the change in recovery. These results indicate that in order to enhance the recovery process, interventions should facilitate engagement in personally meaningful activities and support service users’ possibilities to create control and independence in daily life. Few existing interventions in mental health services today focus explicitly on both these aspects. Occupational therapy would be the nearest existing alternative, but although it has a long history in mental health care, only a few specific manual-based occupational therapy interventions and well-controlled outcome studies exist [21]. Hence, this rehabilitation area should be further developed and researched and also have a prominent place in recovery theory and practice development.

Three possible mediators did not uniquely contribute to recovery change, suggesting that activity satisfaction, level, and balance, as well as psychosocial functioning and symptom severity, are all subsumed within the other variables. This does not deem them uninteresting, but just shows they shared their variation with other covariates. The phenomena behind these factors can constitute interesting topics for future work with the constituents of the two treatments under study.Importantly, one of the possible moderators showed to be significant. The interaction, although not strong, indicates that service users with more activity engagement at the follow up also reported higher recovery at the follow up. Based on this, one can speculate that activity engagement during the follow up period is necessary for the continuous positive development of recovery found in this study. That would be in agreement with previous qualitative research showing that being more active in daily life and participate in the surrounding community were important factors for personal recovery [9, 17, 19].

Although SOT and BEL seemed to enhance recovery equally well in the current study, earlier research comparing BEL and SOT shows that BEL was more effective in several respects, such as improved activity engagement and better psychosocial functioning [22]. Research on BEL so far thus supports that BEL is effective in several respects and can be viewed as a recommended activity-based intervention.

### Methodological considerations

Using the QPR [34] to measure recovery in this study can be seen as a strength, since it aligns well with CHIME, one of the most-used frameworks for personal recovery in mental health services today [10]. Research shows that the QPR incorporates all the concepts in the CHIME framework [8, 44]. Some limitations need to be acknowledged, however. Firstly, it should be mentioned that although the Swedish version of QPR has shown sensitivity to change [36], only the English version has been tested for test-retest stability [34], which may be seen as a limitation of this study. Further, the exact participation rate could not be calculated. The reason for this was use of gatekeepers and dissatisfactory administrative routines when registering non-participants. This weakens the study’s external validity. Also, as expected, there was participant dropout over time, which made this study somewhat underpowered with respect to the SOT group. Another study limitation includes that although this was a longitudinal study, the identified mediators must be seen as aspects of the general improvement the mental health service users reached. This study could thus not demonstrate any clear cause and effect. According to theory, however, recovery is the ultimate criterion for treatment outcome in mental health care [9], and can hardly be viewed as a factor causing the phenomena addressed through the mediators. Finally, one of seven possible moderators, activity engagement, revealed a significant effect, but the fact that several possible interactions were tested makes replication very important.

## Conclusion

The study showed that activity engagement and mastery were the strongest mediators of change in recovery during treatment. These results indicate that in order to enhance the recovery process, interventions within mental health care should facilitate engagement in personally meaningful and desired activities and support the target groups´ possibilities to create control and independence in daily life. Since no support was found for moderators or type of intervention, one can conclude that both of the activity-based occupational therapy based treatments were successful, and they seem to have been equally beneficial for men and women, for people of various ages and for various diagnostic groups when aiming at increasing recovery.

## Data Availability

The datasets used and analyzed during the current study are available from the corresponding author on reasonable request.

## Abbreviations

BEL: Balancing Everyday Life
SOT: Standard occupational therapy
GAF: Global Assessment of Functioning
ICD: International Classification of Diseases
POES: Profiles of Occupational Engagement among people with Severe mental illness
SDO: Satisfaction with Daily Occupations
SDO-OB: Satisfaction with Daily Occupations and Occupational Balance
QPR: The Questionnaire about the Process of Recovery
